# Terlipressin‐induced foot ischemia

**DOI:** 10.1002/ccr3.3570

**Published:** 2020-11-20

**Authors:** Fateen Ata, Ahmed Mohamed Elmudathir Ahmed Osman, Ines Dakhlia, Muhammad Zahid

**Affiliations:** ^1^ Department of Internal Medicine Hamad Medical Corporation Doha Qatar; ^2^ Weill Cornell Medicine Doha Qatar

**Keywords:** adverse drug reaction, hepatorenal syndrome, ischemia, terlipressin

## Abstract

Terlipressin‐induced peripheral ischemia is a rare side effect of the drug, which should be timely identified and treated to prevent permanent necrosis.

## Graphical abstract

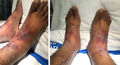

## 1 CLINICAL IMAGE CASE

We present a clinical image of ischemic limbs of a young male with Budd‐Chiari syndrome complicated by hepatorenal syndrome. He was treated with intravenous albumin and terlipressin. The patient developed bilateral foot ischemia, which was promptly identified and reversed by discontinuing the drug and application of glyceryl nitrate patches.

A 27‐years‐old, previously healthy male presented with abdominal pain and distention for ten days. On examination, he was icteric, with moderate ascites. Laboratories showed deranged liver enzymes and high serum creatinine. A diagnostic paracentesis showed serum ascites albumin gradient of 18 g/L. Magnetic resonance cholangiopancreatography (MRCP) findings were suggestive of Budd‐Chiari syndrome. The patient underwent transjugular intrahepatic portosystemic shunt (TIPS) as the patient developed acute liver failure.

**Figure 1:**
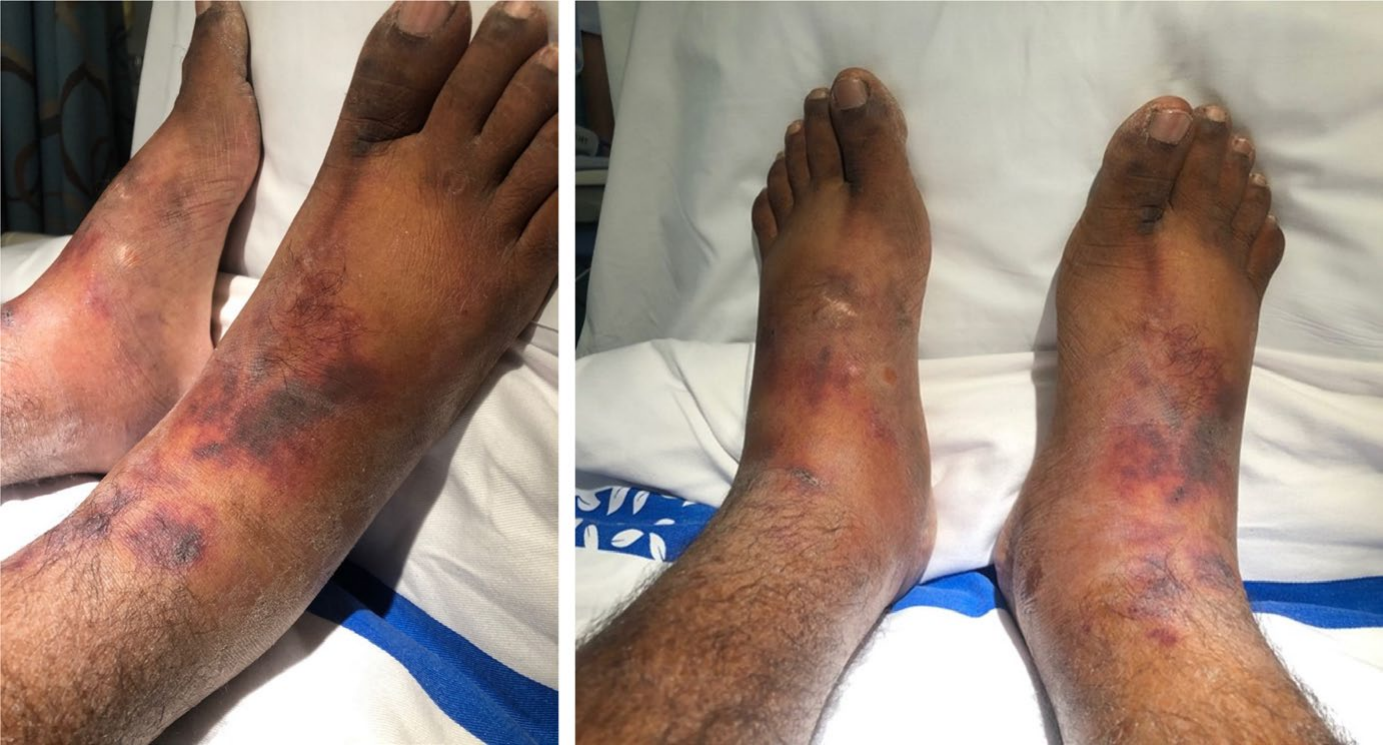
Terlipressin‐induced bilateral lower limb ischemic rash

Patient condition was further complicated by development of Type1 hepatorenal syndrome for which he was commenced on Terlipressin 1mg every 4 hours and albumin solution. Four days later, the patient complained of cramping bilateral foot pain. Examination showed cold extremities, a dark ischemic rash (Figure 1), and a feeble palpable pulse. There was a good triphasic doppler signal over the lower limb arteries. Terlipressin was stopped immediately, and glyceryl nitrate patches were applied to the affected area, leading to symptomatic improvement and resolution of ischemia.

Peripheral ischemia is a rare and debilitating side effect of terlipressin.^1^, ^2^, ^3^ It is reported to develop after 48‐72 hours of initial dose.^1^ Peripheral ischemia must be in the differentials if patients on terlipressin develop limb pains. All efforts should be placed to promptly identify the ischemia and prevent its progression to necrosis, which can be debilitating for the patient.

## CONFLICT OF INTEREST

None declared.

## AUTHOR CONTRIBUTIONS

MZ: involved in conceptualization. FA, AO, ID, and MZ: wrote the manuscript. MZ and FA: selected the image FA, AO, ID, and MZ: reviewed and approved the final manuscript.

## DECLARATION

This manuscript is original work and has not been submitted or is not under consideration for publication elsewhere. All the authors have reviewed the manuscript and approved it before submission. None of the authors have any conflict of interest from publishing this work.

## CONSENT

Written informed consent was obtained from the patient for publication of this case report and accompanying images.

## ETHICS STATEMENT

This work was approved by Medical Research Center (MRC) Qatar before submission.

## Data Availability

Available upon request.
